# Preventative maintenance and unscheduled downtime from an economic perspective

**DOI:** 10.1120/jacmp.v1i2.2647

**Published:** 2000-03-01

**Authors:** Peter Dunscombe, Gisele Roberts, Lianne Valiquette

**Affiliations:** ^1^ Department of Medical Physics Northeastern Ontario Regional Cancer Centre 41 Ramsey Lake Road Sudbury Ontario P3E 5J1

**Keywords:** cancer, radiation, downtime, preventative maintenance

## Abstract

A spreadsheet‐based model for economically characterizing the operation of a radiation treatment program has been used to perform a quantitative financial analysis of scheduled and unscheduled downtime. The incremental cost of downtime is broken down into three categories: remuneration of in‐house or third party service technologists, decreased patient capacity, and local operating procedures for dealing with downtime. Different service arrangements and operating procedures are simulated to demonstrate the financial cost of treatment machine unavailability due to either preventative maintenance or unexpected breakdown. Depending on the service arrangement and operating policies for accommodating downtime, the combined cost of scheduled and unscheduled downtime (at 5%) can exceed 10% of the total cost of the radiation treatment program. It has also been demonstrated that the greatest cost component of downtime is decreased patient capacity, which can exceed $400,000 (CAN) when unscheduled downtime reaches 5%. The interpretation of this cost depends on the funding environment. Although the emphasis of this study has been the financial consequences of downtime, there are other factors which must be considered when developing policies and procedures for accommodating downtime such as effects on treatment, patient convenience and quality of life for staff. Even though the numerical results are strictly valid only within the context of the simulations performed, they do provide a broad framework within which medical physicists can make recommendations regarding service support and downtime. © *2000 American College of Medical Physics.*

PACS number(s): 87.52.–g, 87.90.+y

## I. INTRODUCTION

Among the responsibilities of medical physicists is the safe and reliable operation of radiotherapy treatment and related equipment. Medical physicists are generally primarily responsible for recommending an appropriate service arrangement and advising on operational procedures to be followed in the event of scheduled and unscheduled downtime.^1^ The choices of service arrangement and local operating procedure have significant consequences for the institution including cost, lost real or virtual revenue, patient confidence, and quality of life for the staff. In this paper we have applied an activity costing model of a radiation treatment program (RTP) to the examination of scheduled and unscheduled downtime from an economic perspective. Application of this model allows us to identify the magnitudes of the contributing cost factors and thus places decision making on a more quantitative basis. While the output of simulations such as those described here are dependent on the underlying assumptions and input data, we have provided enough detail for readers to interpret the numerical results in the context of his or her local financial and operating environment.

**Table I:** Utilization pattern for a four megavoltage treatment facility.

	Total per year
Number of patients assessed by Radiation Oncologists	1778
Number of patients who receive external beam radiation therapy (i.e., 90% of those assessed)	1600
Number of patients for whom some type of mould, shield or compensator was customized	1000
Number of patients who were simulated	1600
Number of patients who were CT scanned as part of their preparation activities	256
Number of patients for whom treatment planning by a dosimetrist was required	800
Number of teletherapy fractions administered in the period	28,800
Total hours of operation of the facility	1960

## II. MATERIALS AND METHODS

### A. Model

We have previously developed a sophisticated spreadsheet‐based model for calculating the cost of the various procedures involved in the radiation treatment of cancer.^2^ These include patient assessment, treatment preparation, treatment, and follow up. The model further breaks down the cost of the different activities into appropriate cost categories (i.e., direct labor and materials, administrative overhead, etc.) using accepted health economics principles.^3^

### B. Model inputs

There are four categories of inputs into the model. The first input is workload. This consists of the percentage of time spent by the various professionals performing the activities identified in the radiotherapy process. These input data are based on recommendations made by the professional groups involved and on direct observation. The second input to the model is the financial costs of operating the radiation treatment program such as salaries, capital equipment and overhead costs. These figures are based on 1997 financial data from the Northeastern Ontario Regional Cancer Centre (NEORCC) and are thus in Canadian dollars. Only activities taking place within the program are costed. Administrative infrastructure for the facility as a whole, such as Finance and Human Resources are not included.

The third input is the utilization pattern for a four megavoltage unit treatment facility. Based on a 3.7 fraction per hour capacity per treatment machine and 18 fractions per patient, full utilization of the facility is 1600 patients per year. The numbers of patients undergoing the various procedures identified are based broadly on current practice at NEORCC and are outlined in Table I.

The final input is a standard formula for staffing levels. At the time the project commenced, the recommendations of the Intersociety Council for Radiation Oncology contained in “*Radiation Oncology in Integrated Cancer Management*”^4^ (Blue Book) was an acknowledged, comprehensive, systematic approach to staffing a radiation treatment program. The Blue Book has subsequently been withdrawn but without a replacement. For the purposes of this study we have continued to use Blue Book scaling factors in calculating the number of staff in the various professional groups which constitute the radiation treatment program, Table II.

Table III illustrates in tabular form the level of detail generated by the spreadsheet. OH is an abbreviation for overhead. Utilization figures are those of Table I. With 1600 patients treated annually by this four machine facility, the annual budget for the RTP is $5.4M (CAN). For more information, the reader is referred to Valiquette and Dunscombe.^2^

**Table II:** Staffing formulas for a four megavoltage treatment facility.^4^

Radiation oncologists	$\frac{\text{Patients treated per year}+1\;\text{supervisor}}{250}$	7.5^a^
Nurses	$\frac{\text{Patients treated per year}}{300}$	5.5
Radiation therapists	1 per 12.5 patients treated daily per megavoltage unit+1 supervisor	10.5
Mould room technologists	$\frac{\text{Patients treated per year}}{600}$	3
Simulation room technologists	$\frac{\text{Patients treated per year}\times 2}{500}$	6.5
Physicists	$\frac{\text{Patients treated per year}}{400}$	4
Dosimetrists	$\frac{\text{Patients treated per year}}{300}$	5.5
Electronics technologists and machinists	$\frac{\text{Number of megavoltage units}}{2}$	2
Total staff		44.5^b^

^a^ Rounded up to the nearest 0.5 FTE.

^b^ Plus appropriate support staff.

### C. Downtime assumptions

Scheduled downtime for preventative maintenance is assumed to have two components: extensive preventative maintenance (PM) activities requiring access to the treatment units for 12 hours twice a year and ten minor PMs requiring 4 hours of machine time. These assumptions are based on local practice. Such a schedule has been constant and proved to be adequate over the 8–10 years that our three linacs have been in operation. Quality assurance activities performed by physicists following scheduled downtime are not addressed in these simulations as, under most employment contracts, they do not result in additional or variable costs.

**Table III:** The cost of radiotherapy in a four megavoltage treatment facility treating 1600 patients annually.

	Assessment	Mould prep	Simulation	CT scan	Treatment planning	Teletherapy fraction	Continuing care	Totals
Direct labor costs	$511,184	$218,008	$688,477	$15,487	$374,198	$781,478	$461,544	$3,050,377
Direct material costs	$3,545	$13,330	$12,048	$0	$0	$2,080	$3,191	$34,194
General admin. OH^b^	$50,381	$39,984	$101,644	$3,248	$58,884	$138,439	$45,653	$438,233
Rx Machine OH	$0	$0	$119,868	$0	$100,830	$822,659	$0	$1,043,358
Office & Fixed OH	$92,949	$55,116	$29,247	$0	$20,207	$288,559	$83,654	$569,732
Quality control	$0	$0	$37,332	$0	$12,300	$252,356	$0	$301,987
Total cost per period	$658,059	$326,439	$988,616	$18,735	$566,418	$2,285,572	$594,042	$5,437,881
Utilization	1,778	1,000	1,600	256	800	28,800	1,600	1,600
Total cost per process	$370	$326	$618	$73	$708	$79	$371	
Average cost per patient; i.e., total cost/patients treated per year								$3,399 (CAN)

^a^ Does not include central administration costs such as finance and human resources.

^b^ OH denotes overhead.

The spreadsheet‐based model will, of course, permit the simulation of any amount of downtime. For the purposes of illustration, we have assumed unscheduled downtime to be 5% of scheduled available hours (8 hours per day×245 days=1960 per year), occurring in 1% blocks. It is implicit in the analysis that follows that 8 hours per day for 245 days per year is optimum for the facility. Although we restrict our view to the financial consequences of utilization changes, it is recognized that other factors, e.g., availability of support services, for example psychosocial and dietary counseling, can play a major role in defining “optimum” when used to describe a clinical operation.

### D. Service provision

Three representative service scenarios have been considered:

*Scenario 1.* The institution employs 0.5 of a full time equivalent (FTE) in‐house electronics technologist to perform minor equipment maintenance (minor PMs). An outside service company is utilized for all major PMs and corrective action required for unscheduled breakdowns.

*Scenario 2.* The institution employs 1.5 FTE in‐house electronics technologists for minor equipment maintenance and half of the labor hours spent on unscheduled breakdowns. A service company is employed for the remaining unscheduled breakdown hours and the major PMs.

*Scenario 3.* The institution employs 2.5 FTE in‐house electronics technologists for all minor equipment maintenance, major PMs and unscheduled downtime. There is no outside service support.

In all scenarios, the minor PMs were assumed to be carried out by in‐house staff. Third party service providers are employed at the rate of $300 per hour for labor and travel and are either 1 or 5 hours travel time distant from the facility. The cost of materials is not included in this analysis implying that it would be effectively the same irrespective of service scenarios and would therefore not influence decision making.

## III. RESULTS

The cost of downtime (excluding materials) can be regarded as having three components.

(1) Remuneration of in‐house electronic technologists and/or the third party service company. The option of performing PMs out of hours, with or without overtime premiums, is included.

(2) Decreased patient capacity. One option for dealing with either or both scheduled and unscheduled downtime is to reduce the capacity of the facility in terms of patient numbers in proportion to the number of hours lost to downtime. This cost was obtained by multiplying the average cost per course (Table III) by the number of patients “lost” through downtime minus the small saving in direct materials. In a fee for service funding environment, not fully utilizing the facility represents lost revenue. In a socialized medicine environment, the same dollar figure can be regarded as a “virtual” cost to quantify the underutilization of capital equipment and human resources.

(3) Local operating procedures. In order to avoid reducing the capacity of a facility to account for either or both scheduled and unscheduled downtime, local policy may dictate that lost treatments are made up somehow. A variety of scenarios involving overtime and work schedule changes have been considered and these can lead to extra costs.

**Figure 1:**
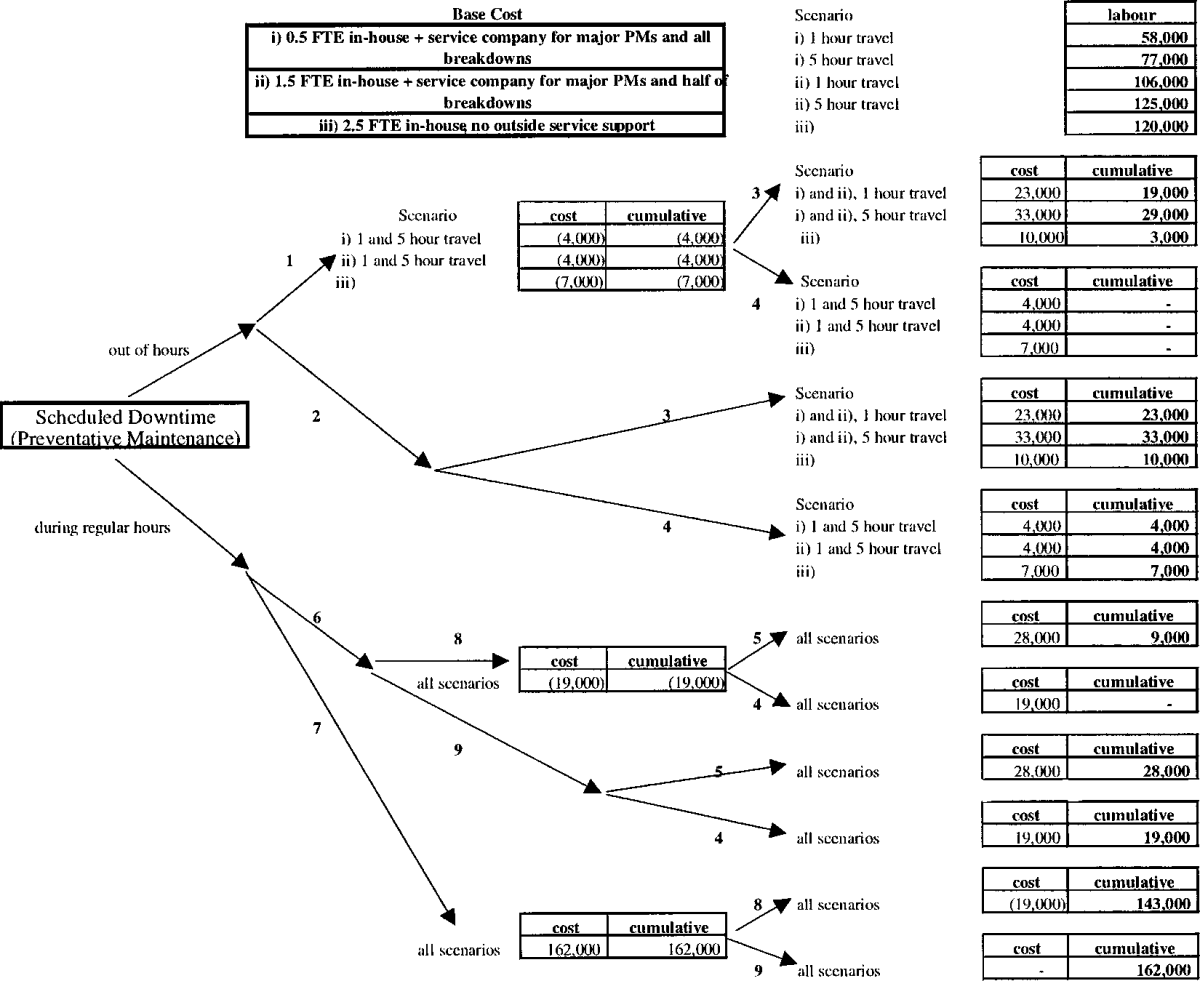
Cost of scheduled downtime. Legend: 1—send electronics technologists home equivalent number of hours during week; 2—electronic technologists work usual hours during week; 3—pay OT to electronic technologists and service company; 4—no OT; 5—pay treatment staff OT; 6–replace treatments; 7—do not replace treatments; 8—send treatment staff home; 9—keep treatment staff. Material and parts costs are not included.

**Figure 2:**
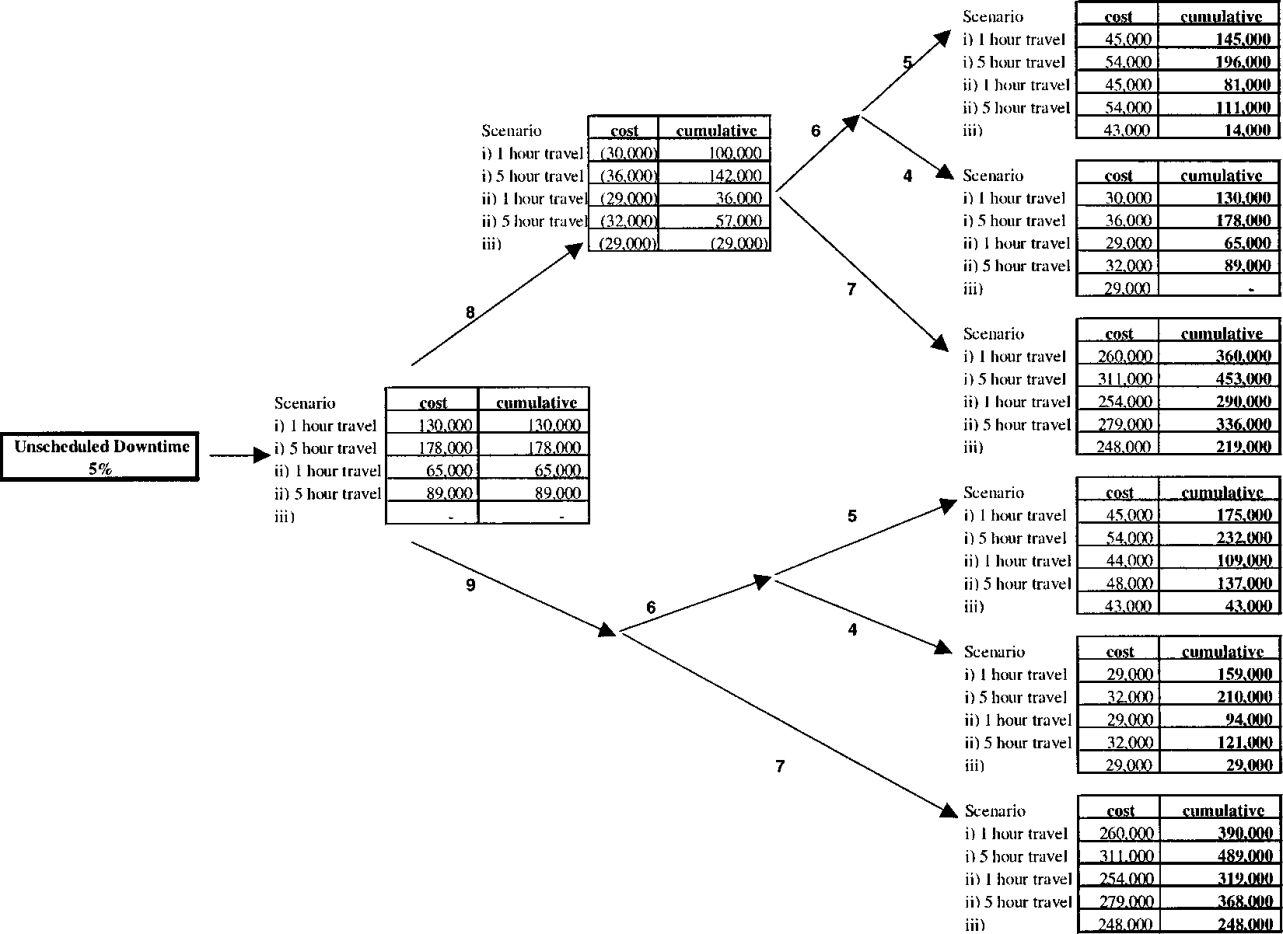
Cost of unscheduled downtime. Legend: 1—send electronics technologists home equivalent number of hours during week; 2—electronic technologists work usual hours during week; 3—pay OT to electronic technologists and service company; 4—no OT; 5—pay treatment staff OT; 6–replace treatments; 7—do not replace treatments; 8—send treatment staff home; 9—keep treatment staff. Material and parts costs are not included.

Figures 1 and 2 show the results of the simulations with all costs rounded up to the nearest $1,000. The total cost of downtime (excluding materials) is the sum of the base cost of the service arrangement (shown at the top of Fig. 1) plus the incremental cost of scheduled downtime, Fig. 1, plus the incremental cost of unscheduled downtime, Fig. 2. For example, the base cost of 1.5 FTE in‐house electronics technologists and third party service of major PMs is $106,000 from the top right hand box of Fig. 1. Of this, $72,000 is salaries and benefits for the in‐house technologists and $34,000 for travel (1 hour each way per PM) and labor (12 hours per machine twice per year) of a third party service company at $300 per hour. If PMs are performed out of hours, then one in‐house service technologist could work correspondingly fewer hours during the normal work week (a “savings” of $4,000, center top box) and the hours would then be made up performing the PMs during non‐treatment times. From the right second box down the cost of having these individuals perform the PMs out of hours is $23,000 if overtime (at 150%) is paid to both in‐house and third party technologists and $4,000 if it is not. The latter is, of course, identical to the savings resulting from the “reduced” workweek discussed above. The former figure of $23,000 is the sum of the payment to the in‐house technologist (at 150%) plus the premium (0.5×$300) paid to the third party service company for 8 12 hour PMs and one hour travel each way. The cumulative columns represent the sums of cost numbers to their left. With PMs performed out of hours and overtime pay, the cumulative cost (excluding materials) is the cost ($23,000) minus the savings ($4,000).

A contrasting situation is that in which no staff are paid overtime, nor sent home, but the treatments which would have been delivered during the PMs are not performed. From the bottom box of the center column of Fig. 1, the lost revenue, or the cost of underutilization of equipment and staff amounts to $162,000.

Handling unscheduled downtime (Fig. 2) is somewhat simpler as, by definition, this refers to interruptions of patient treatments and occurs during regular treatment hours.^5^ To continue with the example of a four machine facility one hour away from a service provider who provides labor for 50% of the unscheduled downtime, the incremental cost of this service is $65,000 (left hand box). If therapy staff are not paid while the equipment is unserviceable, a savings of $29,000 is realized (center top box, Fig. 2). If the therapy staff are then called in to replace the lost treatments and are paid overtime to do so, an additional cost of $45,000 is incurred (right top box). The final cumulative cost (i.e., the total) of 5% unscheduled downtime is, in this case, $81,000 (right top box). In contrast, if the treatments lost due to unscheduled downtime are not replaced, lost revenue would amount to $254,000 for the scenario considered (right bottom box).

In the manner of this example, the total cost of preventative maintenance and 5% unscheduled downtime can be estimated for representative service scenarios and operating procedures.

## IV. DISCUSSION

Clearly the dollar figures which appear in Figs. 1 and 2 are unique to our model, its inputs and assumptions and are not literally transferable to other situations. However, we believe that the trends and ranking of the major cost categories will not change under other realistic inputs and assumptions. The data of Figs. 1 and 2 can therefore be used as the basis for policy decisions in the selection of technical service provision and operating procedures in the event of downtime.

From the results, it can be seen that scheduled PMs and unscheduled downtime of 5% can cost more than 10% ($728,000) of the radiation treatment program budget (excluding spare parts) if a full service contract is held by a facility 5 hours from the service center and lost treatments are not replaced. In contrast, if the same treatment facility relied solely on in‐house electronics staff and employed therapists and other staff who were completely flexible in being rescheduled at no extra cost, the financial consequences of scheduled and unscheduled downtime would be reduced to $120,000.

The largest single component under all scenarios considered is the opportunity cost of lost treatments. This cost varies between $410,000 and $473,000 (by adding the center bottom box of Fig. 1 to the smallest or largest cost figure in the right bottom box of Fig. 2), depending on the service scenario. In a fee for service environment, this represents lost revenue and hence potential profit. In a socialized medicine system, the cost of lost treatments is not recoverable as it is by a change of practice in a fee for service system. However, it does represent an amount that has to be added to an RTP budget which is not linked to output and therefore it can be described as the cost of underutilization of capital and human resources.

The cost of service and therapy staff overtime does not exceed $54,000 (1% of the RTP budget) for any operating procedure considered. Although this is the smallest component of the total cost, as a matter of experience it seems to be the most difficult to justify when asking for funding.

The figures presented here provide a context within which to view other aspects of downtime. If treatments are not replaced and patient numbers are reduced to accommodate scheduled and unscheduled downtime one day of downtime costs up to $5,000, depending on local operating policies and including the cost of lost treatments.

With total labor costs of $150,000 per annum, for example, preventive maintenance is economically justified if 30 machine days of unscheduled downtime are averted or $150,000 worth of premature component failure are avoided or some combination of the two for a four megavoltage unit facility.

Similarly, service contracts with third party organizations can be justified on a financial basis. For example, $100,000 spent on a service contract would be justified if unscheduled downtime is reduced by 2% as a result.

Finally, manufacturers' training courses for electronics personnel can appear, on the surface, to be very expensive. When put in context, however, they could be a very good value. A $20,000 training course is justified on financial grounds if the knowledge so acquired results in just four days of unscheduled downtime being averted.

The subject of this work has been the financial consequences of service arrangements and local operating procedures. Clearly there are other consequences of decisions regarding equipment maintenance. When scheduling preventative maintenance it will be necessary to consider the radiobiological impact of an additional break in treatment; patient convenience; access to component supplies and quality of life for staff, amongst other issues. In reviewing service contracts it has to be recognized that in general a third party organization has both a larger pool of technical expertise and a more comprehensive parts inventory than can be held in one treatment facility.

## V. CONCLUSIONS

We have presented a quantitative financial analysis of scheduled and unscheduled downtime. As emphasized above, the exact numbers generated and presented in Figs. 1 and 2 depend on the input financial data to the model and the assumptions made, about which we have been explicit. Although our numerical results are only strictly valid within the context of our simulations, they provide a broad framework within which to make decisions regarding service support and downtime. From the authors' observations, most radiation treatment programs operate in a manner close to that used as the basis of the model and thus the ranking and relative magnitudes of the cost components studied are generally valid.

## ACKNOWLEDGMENTS

The authors gratefully acknowledge the Northern Cancer Research Foundation for financial support of this project.
